# Identification of genes related to tipburn resistance in Chinese cabbage and preliminary exploration of its molecular mechanism

**DOI:** 10.1186/s12870-021-03303-z

**Published:** 2021-12-03

**Authors:** Jingping Yuan, Changwei Shen, Ranghua Yuan, Huaixia Zhang, Yan Xiao, Xiaoling Wang, Feifei Pan, Chunhui Wu, Qingfei Li, Jingyun Yuan, Xuesheng Liu

**Affiliations:** 1 School of Horticulture and Landscape Architecture, Henan Institute of Science and Technology, Xinxiang, 453003 China; 2 Henan Engineering Research Center of the Development and Utilization of Characteristic Horticultural Plants, Xinxiang, 453003 China; 3 School of Resources and Environmental Sciences, Henan Institute of Science and Technology, Xinxiang, 453003 China; 4 Vegetable Research Institute of Xinxiang Academy of Agricultural Sciences, Fifty Meters Southwest of the Intersection of Xiner Street and Rongxiao East Road, Hongqi District, Xinxiang City, 453003 Henan Province China

**Keywords:** Chinese cabbage, Tipburn, Transcriptome sequencing, Differentially expressed genes (DEGs)

## Abstract

**Background:**

Tipburn, also known as leaf tip necrosis, is a severe issue in Chinese cabbage production. One known cause is that plants are unable to provide adequate Ca^2+^ to rapidly expanding leaves. Bacterial infection is also a contributing factor. Different cultivars have varying degrees of tolerance to tipburn. Two inbred lines of Chinese cabbage were employed as resources in this research.

**Results:**

We determined that the inbred line ‘J39290’ was the tipburn resistant material and the inbred line ‘J95822’ was the tipburn sensitive material based on the severity of tipburn, and the integrity of cell membrane structure. Ca^2+^ concentration measurements revealed no significant difference in Ca^2+^ concentration between the two materials inner leaves. Transcriptome sequencing technology was also used to find the differentially expressed genes (DEGs) of ‘J95822’ and ‘J39290’, and there was no significant difference in the previously reported Ca^2+^ uptake and transport related genes in the two materials. However, it is evident through DEG screening and classification that 23 genes are highly linked to plant-pathogen interactions, and they encode three different types of proteins: CaM/CML, Rboh, and CDPK. These 23 genes mainly function through Ca^2+^-CaM/CML-CDPK signal pathway based on KEGG pathway analysis, protein interaction prediction, and quantitative real-time PCR (qRT-PCR) of key genes.

**Conclusions:**

By analyzing the Ca^2+^ concentration in the above two materials, the transcription of previously reported genes related to Ca^2+^ uptake and transport, the functional annotation and KEGG pathway of DEGs, it was found that Ca^2+^ deficiency was not the main cause of tipburn in ‘J95822’, but was probably caused by bacterial infection. This study lays a theoretical foundation for exploring the molecular mechanism of resistance to tipburn in Chinese cabbage, and has important guiding significance for genetics and breeding.

**Supplementary Information:**

The online version contains supplementary material available at 10.1186/s12870-021-03303-z.

## Background

Chinese cabbage (*Brassica rapa* L. ssp. *pekinensis*), a member of *Cruciferae*, has a long history of cultivation and has the laudatory name of “king of vegetables”. In the winter and spring, it is also the most popular vegetable on people’s tables in North and Northeast China, and it has a large planting area. According to statistics, the annual 50 sown area of Chinese cabbage reaches 2.67 million hm2, accounting for 15% of the total sown area of vegetables in the country, with an output value of nearly 60 billion Yuan (RMB) [1, 2]. Today, vast regions of cultivation can be found in Japan, Southeast Asia, Europe, and the United States, and it has become an inseparable delicacy on people’s dinner tables all over the world. People prefer Chinese cabbage because it has a wide range of adaptation, can be widely planted, has rich nutrition, variety diversity, high yield and storage tolerance, and is high in carotenoids, protein, crude fibre, and other nutrients.

Tipburn of Chinese cabbage is a physiological disorder that has gained a lot of attention in recent years. It’s symptom is a dry and yellow edge to the interior leaf at the rosette stage, which wilts in a white band following dehydration, and some young leaves have a dry edge. The typical plant tipburn incidence rate of Chinese cabbage is 10-20%, and in severe circumstances, it can reach as high as 80% [3]. Farmers have suffered significant economic losses as a result of Chinese cabbage tipburn, which is getting increasingly severe. Tipburn starts in the rosette stage and becomes more serious as it progresses to the heading stage [3, 4].

It has always been studied that the occurrence of tipburn is mainly due to calcium deficiency [5, 6]. Some researchers believe that tipburn is caused by a complex interaction of elements such as impaired calcium uptake and transport, an aberrant growing environment, and uncoordinated growth, rather than by a shortage of calcium in the soil [7].

In response to diverse stress signals, Ca2+ homeostasis in the cytosol was significantly altered in plants [8–10]. Intracellular Ca2+ homeostasis is controlled by Ca2+ storage and transport system [11]. Ca2+ is mainly stored in plasma membrane, vacuole and endoplasmic reticulum [10]. The most typical Ca2+ transporters are ACA (Autoinhibited Ca2 + −ATPase), CAX (Ca2+/H+ antiporter) located in vacuoles and ECA (Endoplasmic reticulum-bound Ca2 + pump) protein located in endoplasmic reticulum [10, 12, 13].

In Chinese cabbage, seven *ECA* genes and four *CAS* (Calcium sensor) genes were discovered, and their expression was influenced by calcium stress, which might be linked to the incidence of tipburn at the seedlings stage [14]. Besides, the expressions of *ECA*, *ACA* and *CAS* in inner, middle and outer leaves of different lines were compared by RT-PCR. The results showed that the lack of calcium transport in the inner leaves is the key to the occurrence of tipburn. Some circRNAs may be involved in the response of plants to calcium deficiency induced tipburn through the circRNA regulation pathway [15]. Su et al. [16] found that *BrCRT2* is an important gene for tipburn resistance in Chinese cabbage. Despite the discovery of several genes linked to tipburn resistance in Chinese cabbage, 84 the mechanism of tipburn resistance remains unknown.

At present, in addition to calcium deficiency causing tipburn in Chinese cabbage, it has also been found that pantomime *P antoea ananatis* can cause bacterial tipburn in Chinese cabbage [17]. Biological characteristics of *P.ananatis Y2* strain were analyzed and its whole genome was sequenced, which is helpful to accelerate the study of tipburn in Chinese cabbage, and is of great significance to fully reveal the molecular mechanism of tipburn in Chinese cabbage and to cultivate high quality, high yield and disease resistance Chinese cabbage varieties.

Zhang et al. (1994) [18] found that there were differences in the sensitivity of different varieties of Chinese cabbage to tipburn. Although the resistance of Chinese cabbage to tipburn is determined by genetic factors, it is controlled by polygenes and belongs to quantitative traits [6]. At present, the research on tipburn is mainly focused on field prevention and inducing factors, but there are few reports on the molecular mechanism of the tipburn [19]. This work employed tipburn resistant plants and sensitive inbred lines as materials to uncover and evaluate tipburn related genes in Chinese cabbage in order to systematically understand its molecular mechanism. It has guiding significance and application value in analyzing the molecular mechanism of tipburn response of Chinese cabbage and improving stress resistance.

## Results

### Phenotypic analysis of two Chinese cabbage inbred lines—‘J95822’ and ‘J39290’

**Fig. 1:**
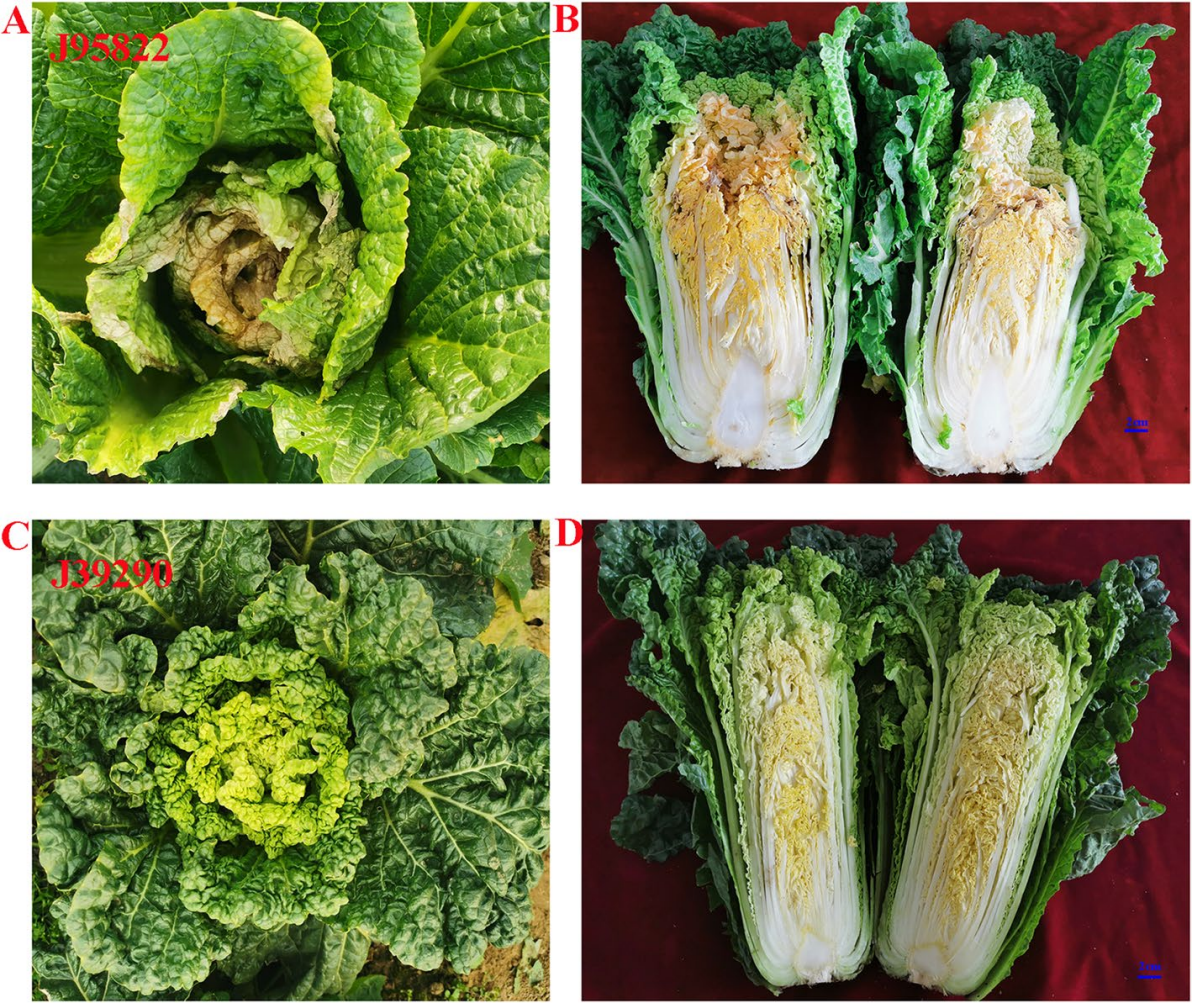
Symptoms observation on tipburn in Chinese cabbage during heading stage. **A** Top view of inbred line ‘J95822’; **B** Longitudinal section section of inbred line ‘J95822’; **C** Top view of inbred line ‘J39290’; **D** Longitudinal section profile of inbred line ‘J39290’. Bar: 2 cm

We studied two inbred line i.e.,‘J95822’ and ‘J39290’, to see how Chinese cabbage responded to tipburn. We discovered that ‘J95822’ and ‘J39290’ had similar leaf shape and color in the field by monitoring the phenotypic of two homozygous lines at heading stage. The leaves of ‘J95822’, on the other hand, showed clear tipburn symptoms (Fig. 1A), especially the interior leaves, which had dried out (Fig. 1B), but the leaves of ‘J39290’ did not (Fig. 1C and D). As a result, ‘J95822’ is a tipburn sensitive line, while ‘J39290’ is a tipburn resistant line.

### Incidence rate of tipburn in two Chinese cabbage inbred lines—‘J95822’ and ‘J39290’

**Fig. 2:**
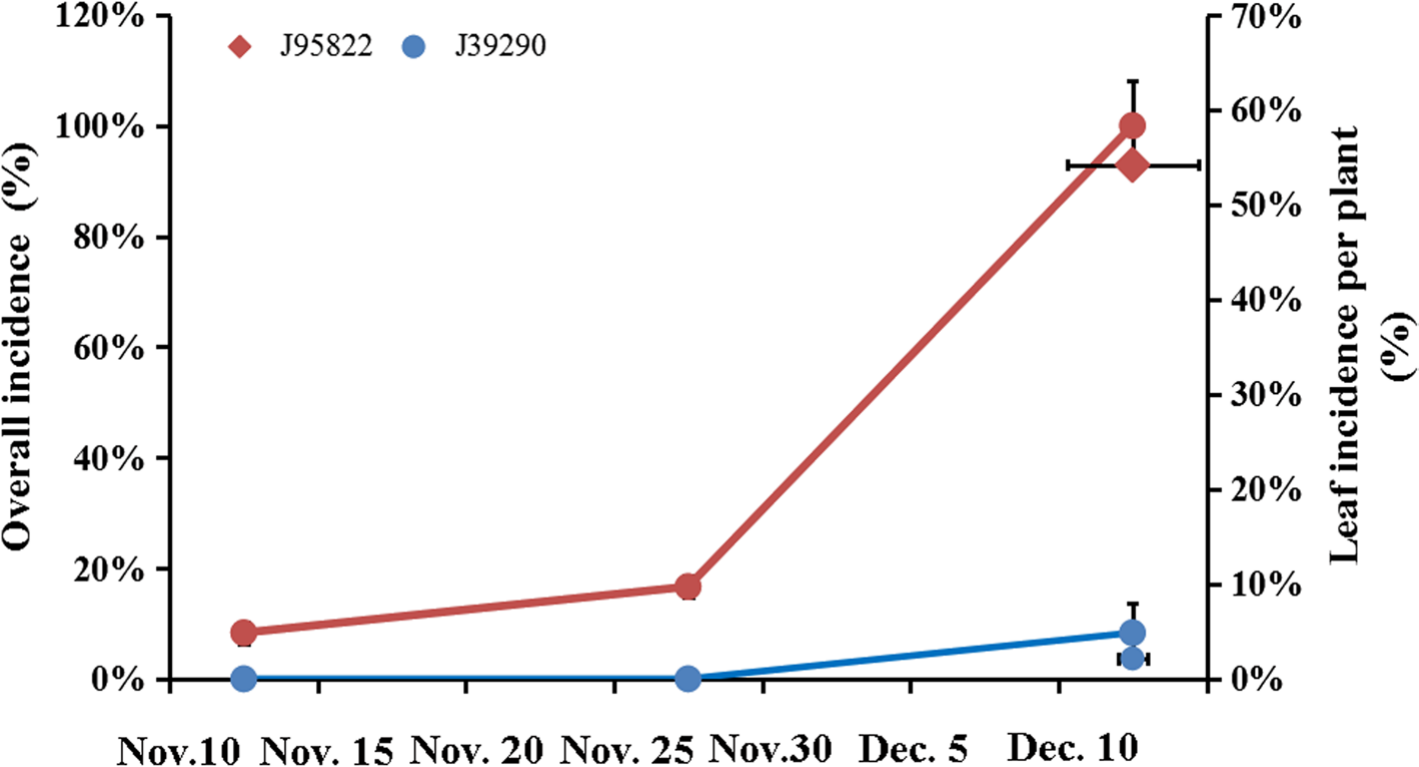
Statistics on the overall tipburn incidence of inbred lines ‘J95822’ and ‘J39290’ at rosette stage, early heading stage, late heading stage and leaf tipburn incidence per plant at late heading stage. The abscissa is the date, the left ordinate is the overall incidence, and the right ordinate is the leaf incidence per plant. Nov. 12, the rosette stage; Nov.27, the early heading stage; Dec.12, the late heading stage. Twelve plants were selected for each variety

We evaluated the overall tipburn incidence of Chinese cabbage varieties ‘J95822’ and ‘J39290’ during the rosette and heading stages, respectively. The results revealed that the overall tipburn incidence of ‘J95822’ was 8.33% in the rosette stage, while that of ‘J39290’ was 0 (Fig. 2). The overall tipburn incidence of ‘J95822’ was 16.67% in the early heading stage, while that of ‘J39290’ was 0. In the late heading stage, the leaf tipburn incidence per plant in ‘J95822’ plants was 100%, while that of ‘J39290’ was 8.33% (Fig. 2). In the late heading stage, the leaf tipburn incidence per plant in ‘J95822’ plants was 100%, while that of ‘J39290’ was 8.33% (Fig. 2). Through the statistics of leaf tipburn incidence per plant, we found that there was no significant difference in the total leaf number between ‘J95822’ and ‘J39290’, both in the range of 24-34 leaves. The number of tipburnd leaves per plant was also counted during the late heading stage. Only 2.15% of the leaves in ‘J39290’ had tipburn symptoms, while 54.19% of the leaves in ‘J95822’ had tipburn symptoms (Fig. 2). We infer that ‘J95822’ is a tipburn sensitive line and ‘J39290’ is a tipburn resistant line based on the aforesaid analysis.

### Microstructure of interior leaves of tipburn sensitive and tipburn resistant Chinese cabbage

**Fig. 3:**
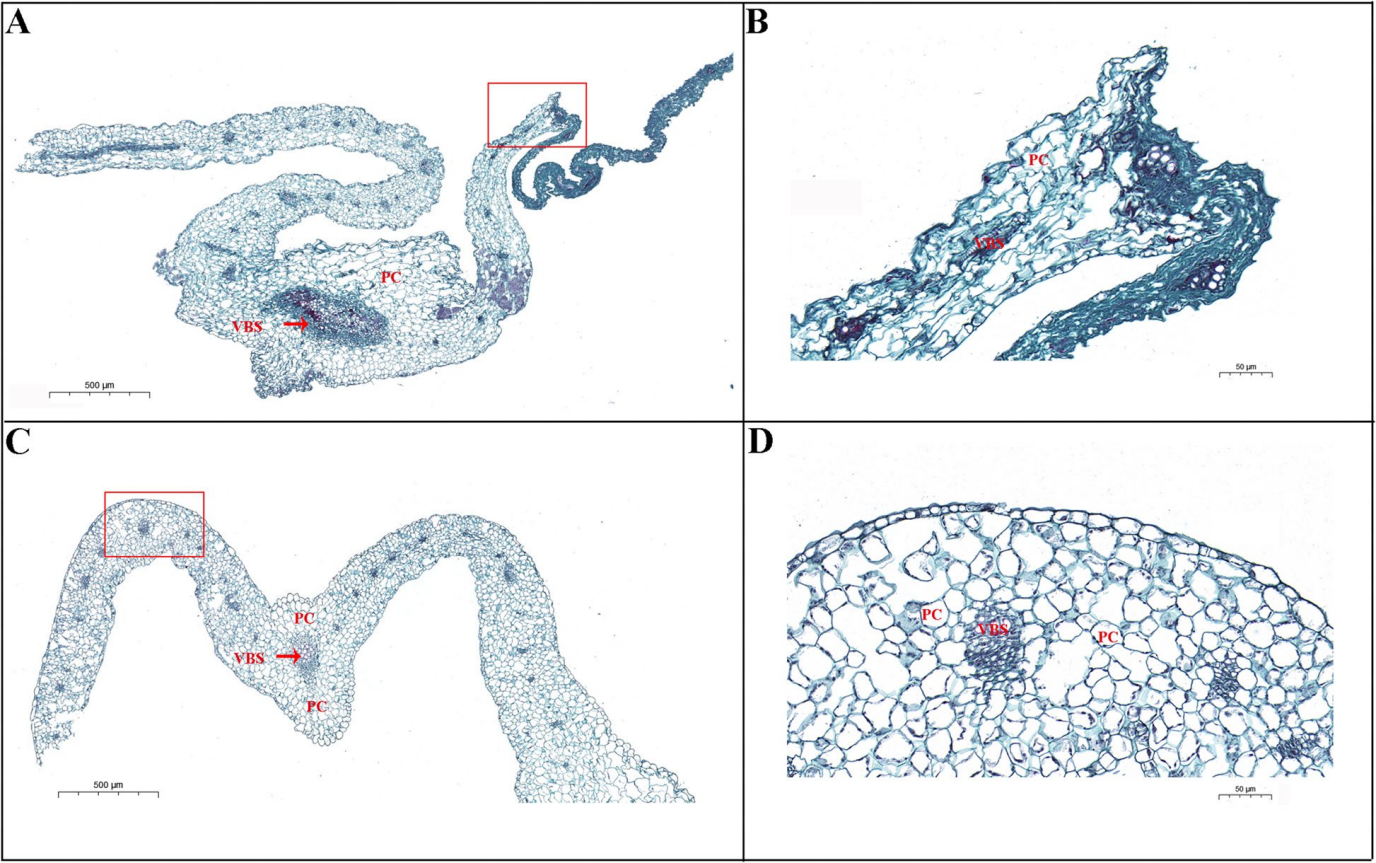
Microstructure of interior leaves of tipburn sensitive plant ‘J95822’ and tipburn resistant plant ‘J39290’. **A** and **B** cellular structure of tipburn sensitive Chinese cabbage ‘J95822’. **C** and **D** cellular structure of tipburn resistant Chinese cabbage ‘J39290’. Figures **B** and **D** are local magnification of figures **A** and **C**, respectively. PC: parenchyma cell; VBS: vascular bundle sheath. Bar: 500 μm (**A**, **C**); 50 μm (**B**, **D**)

We investigated the micro-anatomical structure of the tipburn site (interior leaf) of ‘J95822’ and ‘J39290’, to see if there were any variations in cell structure between tipburn sensitive and resistant Chinese cabbage varieties (Fig. 3A and C). The interior leaf cell structure of ‘J95822’ and ‘J39290’ was essentially the same, but the parenchyma cells of ‘J95822’ had a serious damage phenomenon at the site of onset (Fig. 3B), whereas the vascular bundle sheath of interior leaf of ‘J39290’ was obvious, and the parenchyma cells were regular in shape and uniform in size (Fig. 3D). Therefore, we speculated that the tipburn of Chinese cabbage mainly destroyed the integrity of cell membrane of interior leaves.

### Nutrient analysis in roots and leaves of tipburn sensitive and tipburn resistant Chinese cabbage

**Fig. 4:**
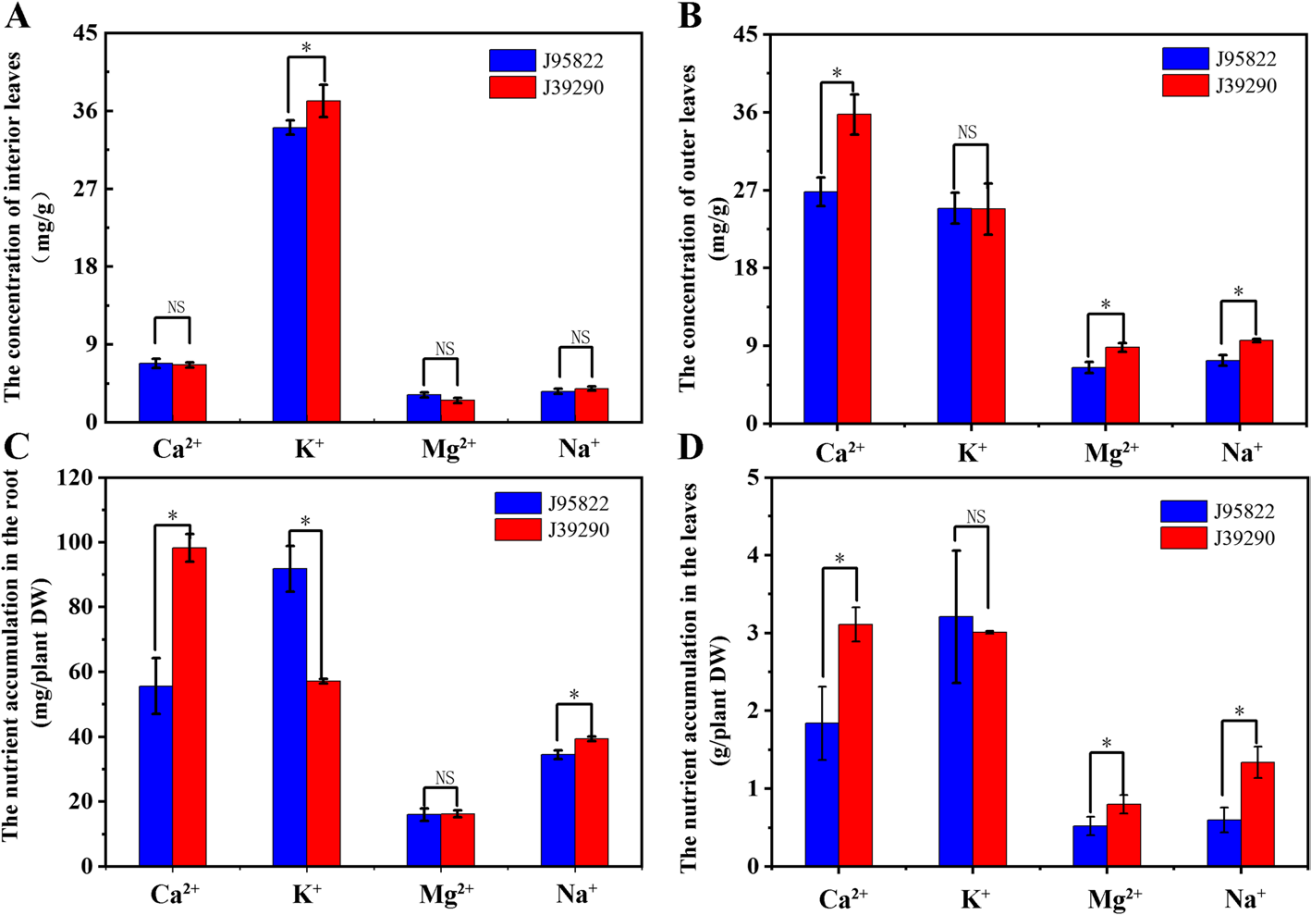
Nutrient content of tipburn sensitive plant ‘J95822’ and tipburn resistant plant ‘J39290’. **A** Nutrient concentration in the interior leaves of tipburn sensitive plants and tipburn resistant plants. **B** Nutrient concentration in the outer leaves of tipburn sensitive plants and tipburn resistant plants. **C** Nutrient accumulation in roots of tipburn sensitive plants and tipburn resistant plants. **D** nutrient accumulation in interior leaves of tipburn sensitive plants and tipburn resistant plants. Each data represents the average from three samples. The error bars represent the SDs. ∗ indicates significance at *p* < 0.05, ∗ ∗ indicates significance at *p* < 0.01, NS indicates no significance

Considering ion uptake and transport play an important role in plant resistance to stress, we analyzed the contents of Ca2+, K+, Mg2+ and Na + in roots, leaves of tipburn sensitive ‘J95822’ and tipburn resistant ‘J39290’. The accumulation of K+ in root from ‘J39290’ (56.50-57.80 mg/plant DW) was lower than ‘J95822’ (84.75-98.87 mg/plant DW) (Fig. 4C), but there was no significant difference in interior leaves (Fig. 4D). The accumulation of Mg2+ in the root of ‘J39290’ did not differ significantly from that of ‘J95822’ (Fig. 4C), whereas the accumulation of Mg2+ in the interior leaf of ‘J39290’ was higher than that of ‘J95822’. The Mg2+ concentration in the outer leaves of ‘J39290’ was significantly higher than that of ‘J95822’ (Fig. 4B). The results revealed that between tipburn susceptible and tipburn resistant Chinese cabbage, ion uptake and transport differed. In terms of calcium accumulation, Ca2+ accumulation in roots of ‘J39290’ was 1.46 ~ 2.18 times higher than that of ‘J95822’ (Fig. 4C), and Ca2+ accumulation in interior leaves of ‘J39290’ was 1.25 ~ 2.44 times higher than in interior leaves of ‘J95822’ (Fig. 4D). In terms of Ca2+ concentration, ‘J39290’ increased by 4.94-12.89 mg/g in the outer leaves compared to ‘J95822’ (Fig. 4B), but there was no significant difference in the interior leaves (Fig. 4A). Given that calcium uptake and transport affect plant tipburn, yet there was no significant difference in Ca2+ concentration in the interior leaves of the two inbred lines, we speculate that calcium deficiency is not the primary cause of tipburn, while bacterial tipburn may be present.

### Transcriptome sequencing of interior leaves of tipburn sensitive and tipburn resistant Chinese cabbage

**Fig. 5:**
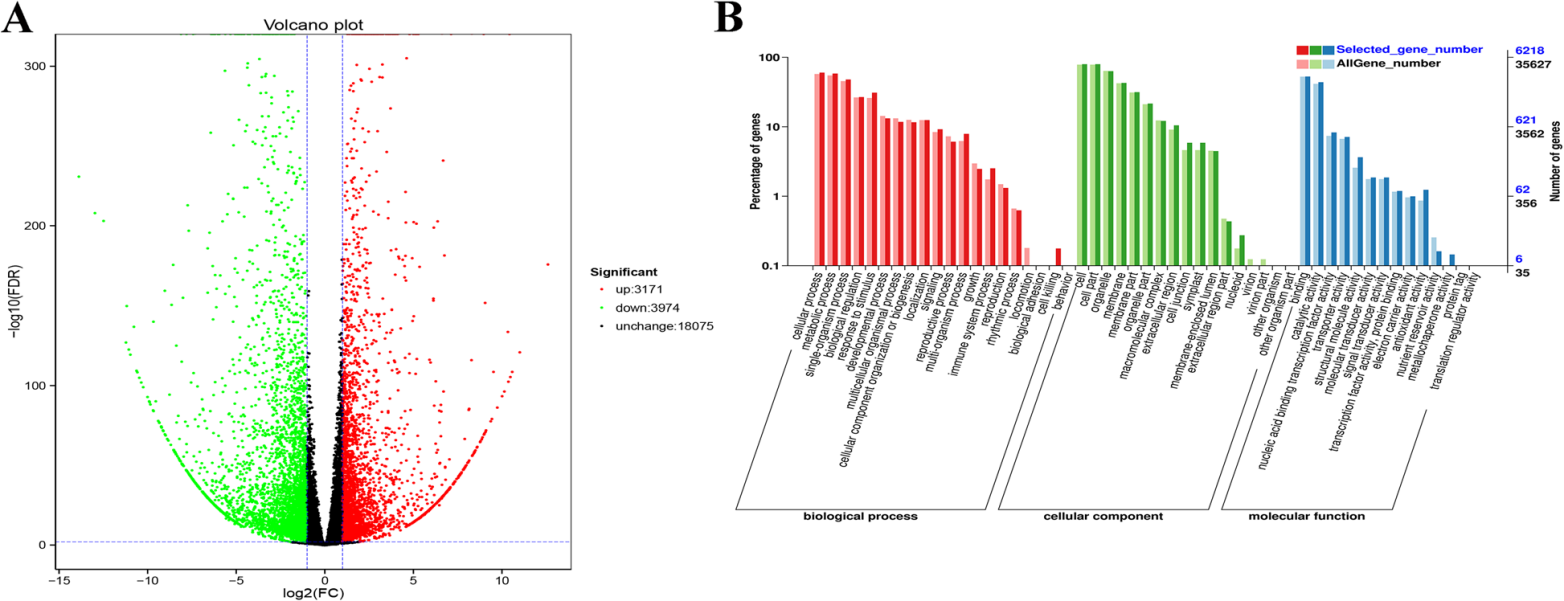
Screening of DEGs between tipburn sensitive material ‘J95822’ and tipburn resistant material ‘J39290’. **A** Volcano plot of DEGs. Red dots represent up-regulated DEGs, green dots represent down-regulated DEGs, and black dots represent non-differentially expressed genes. **B** GO annotation classification statistics of DEGs. The abscissa is the GO classification, the ordinate on the left is the percentage of the number of genes in the total number of genes, and the right is the number of genes

A total of 42.76 GB clean data was collected from transcriptome sequencing of the interior leaves (6 samples) from ‘J95822’ and ‘J39290’ during the heading stage. Each sample’s clean data totaled 6.24 GB, with Q30 bases of 93.27% and higher (Additional file 1: Table S1).The alignment efficiency of the clean reads with the designated Chinese cabbage genome (Bra Chromosome V1.5) ranged from 85.22 to 88.24% (Additional file 1: Table S2), indicating that the use rate of transcriptome data was extremely high. A total of 42,317 genes, including 2086 novel genes, were found by splicing and functional annotation of mapped data. Through principal component analysis (PCA) of all genes in ‘J95822’ and ‘J39290’, it was found that PCA1 and PCA2 were 87.5 and 11.3%, respectively (Additional file 2: Fig. S1). The biological repeats of each material were significantly clustered together (Additional file 2: Fig. S1), indicating that the transcriptome sequencing results were reliable. In this study, with the tipburn sensitive samples ‘J95822’ as the control, a total of 7145 DEGs were discovered in the tipburn resistance samples ‘J39290’. Volcano plot showed that 3171 genes were up-regulated and 3974 genes were down-regulated (Fig. 5A). Through gene ontology (GO) analysis of all DEGs, they contained three main branches, namely: biological process, molecular functional and cellular component. They were mostly involved in the cellular process, cell, cell membrane components and binding function of biological processes (Fig. 5B).

### Screening of candidate genes related to tipburn

**Fig. 6:**
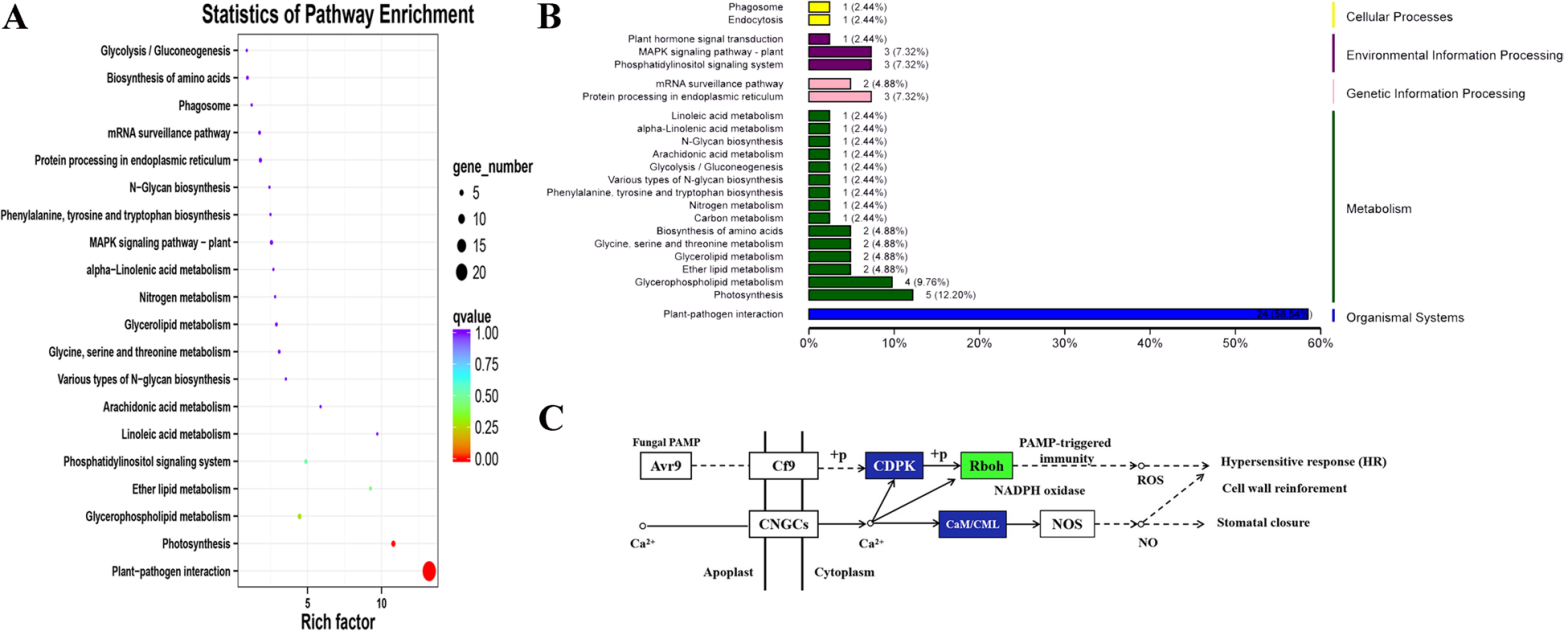
Screening and analysis of genes related to tipburn. **A** KEGG enrichment map of 117 tipburn related genes; **B** KEGG classification map of 117 tipburn related genes; **C** KEGG pathway of 23 calcium related genes. Genes with blue background indicate that the expression level of genes encoding these proteins are up-regulated and down-regulated. The genes with green background indicate that the expression level of genes encoding these proteins are down-regulated

The transcriptome data of the interior leaves of the tipburn resistant plant ‘J39290’ were extracted using the interior leaves of the tipburn sensitive plant ‘J95822’ as a control. Total of 117 DEGs (2% of all DEGs) associated with Ca2+ were found by gene functional annotation, among which there were no previously reported genes associated with Ca2+ uptake and transport (Additional file 1: Table S5). However, 24 genes were shown to be involved in plant-interactions, according to the KEGG enrichment map (Fig. 6A, B). Twenty three of these genes were involved in hypersensitive response, cell wall reinforcement, and stomatal closure, among other functions (Fig. 6C). Seven genes were up-regulated and 16 genes were down-regulated (Table 1; Additional file 1: Table S3), and they encoded three types of proteins: CDPK, Rboh, and CaM/CML (Table 1; Additional file 1: Table S3). We speculated that these genes regulate tipburn resistance via the calcium signal, based on the aforementioned analysis. We predicted that by interacting with pathogens, these genes regulate tipburn resistance so that ‘J39290’ could improve its tipburn resistance.

**Table 1:** Expression information of 23 genes related to tipburn in sensitive and resistant materials

Gene ID	Protein_name^*1^	FDR^*2^	log_2_FC^*3^	Regulated^*4^
Bra002099	BrCDPK16	1.12E-76	1.958996331	up
Bra003287	BrCDPK12	2.20E-86	−1.38085198	down
Bra009653	BrCDPK6	6.11E-99	−2.69335857	down
Bra012889	BrCML41	2.61E-76	−1.81948223	down
Bra013575	BrCDPK15	3.93E-55	−2.74059595	down
Bra015727	BrCML38	1.67E-33	−3.50673612	down
Bra015979	BrCML26	3.95E-35	−2.41390299	down
Bra017355	BrCML16	7.96E-29	−1.51636801	down
Bra017707	BrCDPK5	1.43E-36	−7.08563893	down
Bra019189	BrRboh	2.01E-21	−5.1675852	down
Bra019503	BrCML47	1.01E-05	−2.53644532	down
Bra019554	BrCML5	2.07E-46	4.102775767	up
Bra023786	BrCML11	1.86E-24	1.503165036	up
Bra025439	BrCML24	2.75E-92	1.283979067	up
Bra027981	BrCML37	7.97E-11	−2.7771056	down
Bra028360	BrCML22	5.96E-16	−3.41148864	down
Bra029378	BrCDPK12	1.06E-26	2.91831559	up
Bra032785	BrCML25	9.86E-06	1.747227016	up
Bra033077	BrCML45	7.98E-18	−1.86427236	down
Bra034103	BrCML49	4.46E-281	−2.99553328	down
Bra035972	BrCML5	6.39E-19	1.273893559	up
Bra037277	BrCDPK6	1.70E-33	−1.73271106	down
Bra039511	BrCML43	1.34E-13	−4.14267425	down

^*1^ The protein name obtained through functional annotation

^*2^ FDR: false discovery rate

^*3^ Log_2_FC: the logarithm of differential expression fold change of differentially expressed genes;

^*4^ Regulated: up-regulated gene (up) or down-regulated gene

### qRT-PCR determination of genes related to tipburn

**Fig. 7:**
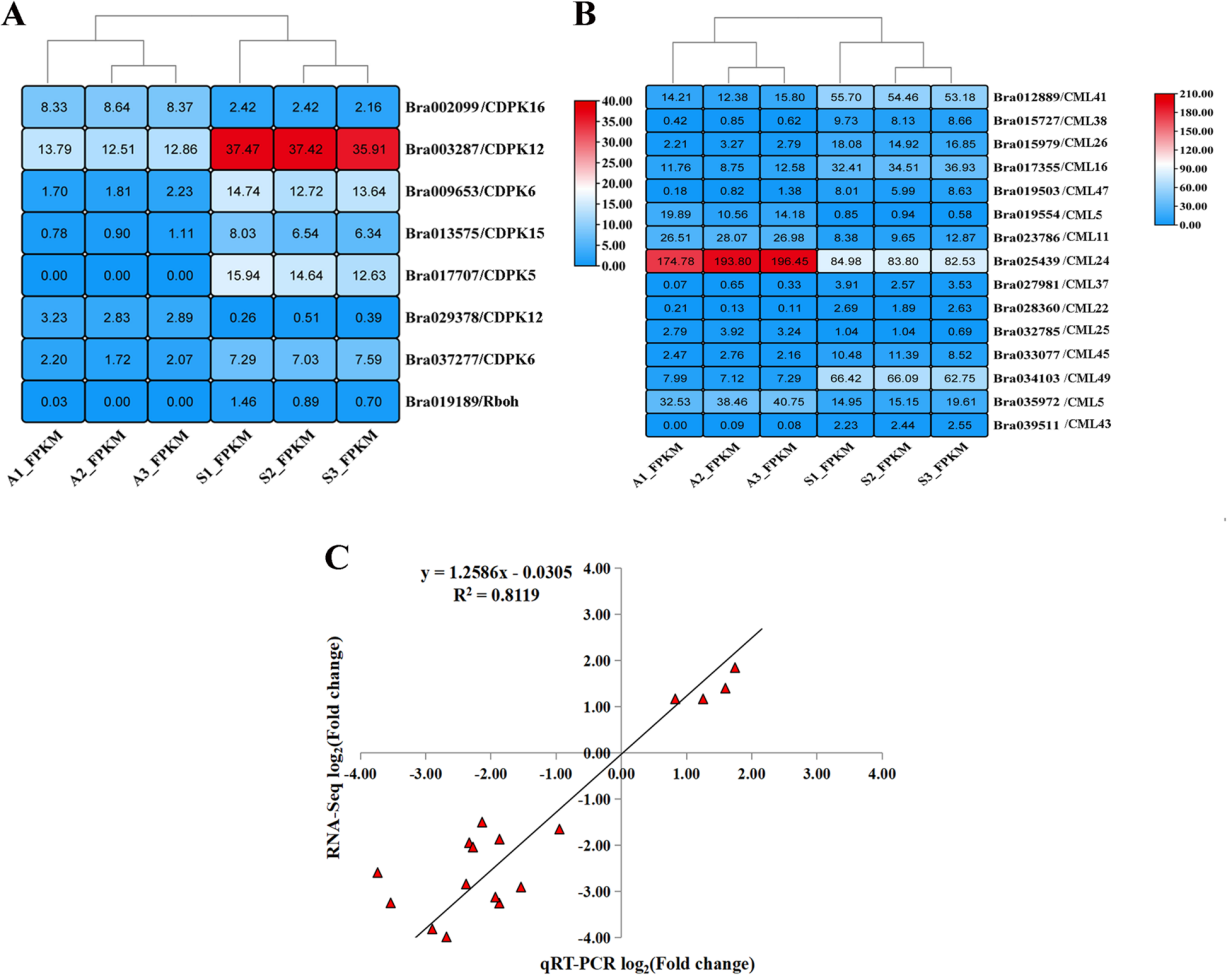
Heat map and cluster analysis of FPKM data of 23 tipburn related protein genes and their correlation with qRT-PCR data. **A** Heat map and cluster analysis of FPKM data of 7 CDPK protein genes and 1 Rboh protein gene (Gene ID: Bra019189); **B** Heat map and cluster analysis of FPKM data of CaM/CML protein genes. The value in the heat map indicates the FPKM value of the gene. Three samples of tipburn resistant interior leaves were named A1, A2 and A3 respectively, and three samples of tipburn sensitive interior leaves were named S1, S2 and S3, respectively. **C** The correlation analysis between FPKM value and qRT-PCR value of 21 tipburn related protein genes. “*R2*” represented pearson correlation coefficient

Cluster analysis of the FPKM data of 23 tipburn-related protein genes showed that there was a strong correlation between the biological repeats of tipburn sensitive and tipburn resistant samples (Fig. 7). There were 5 genes that were down-regulated and 2 genes that were up-regulated among the *BrCDPK* genes, with the log2FC of *Bra017707* (*BrCDPK7*) being − 7.08 and the log2FC of *Bra029378* (*BrCDPK12*) being 2.91. Moreover, *BrRboh* gene (Gene ID: *Bra019189*) was significantly down-regulated (Fig. 7B). Among the *BrCaM*/*BrCML* genes, there were 10 genes down-regulated and 5 genes up-regulated, of which the log2FC of *Bra019554* (*BrCML5*) gene was 4.10, and the *Bra039511* (*BrCML43*) was − 4.14 (Fig. 7B, Additional file 1: Table S3).

**Fig. 8:**
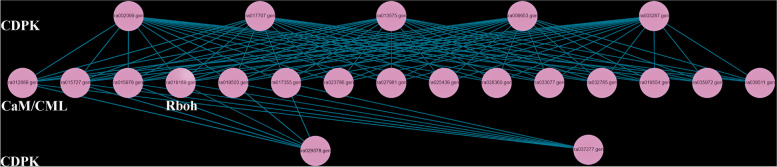
Interaction map of 23 proteins related to tipburn

To validate the transcriptomic data using qRT-PCR, a total 23 candidate genes were selected. The results showed that 6 genes were up-regulated, 16 genes were down-regulated (Additional file 2: Fig. S2), and 1 gene (*BrCDPK12*-2) was not expressed. In addition, we found that the expression levels of *Bra012889* (*BrCML41*) and *Bra003287* (*BrCDPK12-1*) genes in tipburn resistant samples were 0.2 and 0.23 times that of tipburn sensitive plants, respectively. The expression levels of *Bra019554* (*BrCML5-1*) and *Bra025439* (*BrCML24*) in tipburn resistant samples were 4.45 and 1.17 times higher than those of tipburn sensitive plants, respectively. The pearson correlation coefficient (*R2*) was used to examine the correlation between transcriptome data and qRT-PCR data, and the results showed that the *R2* was 0.81, indicating a positive correlation between the RNA sequencing data and qRT-PCR data (Fig. 7C). Based on the findings, we infer that, in addition to *BrCDPK12-2*, the other 22 candidate genes are important in tipburn stress.

### Interaction analysis of proteins encoded by 23 tipburn candidate genes

The prediction of protein interaction showed that Bra034103 (CML49) did not interact with any proteins, while Bra012889 (CML41), Bra015727 (CML38), Bra015979 (CML26), Bra017355 (CML16) and Bra019503 (CML47) interacted with all CDPK proteins. While, the remaining 9 CaM/CML proteins (Bra019554, Bra023786, Bra025439, Bra027981, Bra028360, Bra032785, Bra035972, Bra039511, Bra033077) interacted with 5 CDPK proteins (Bra002099, Bra003287, Bra009653, Bra013575, Bra017707). All CDPK proteins interact with Rboh proteins. These findings are in line with the KEGG pathway. Based on the findings, we hypothesized that, as compared to the inbred line ‘J95822’, ‘J39290’ may improve tipburn resistance in inner leaves via the Ca2 + −CaM / CML-CDPK signal pathway (Fig. 8).

## Discussion

Tipburn, commonly known as skin rot, is a physiological disorder that affects Chinese cabbage during the rosette and heading stages. The majority of the injured leaves are in the middle of the leaf bulb, and an injured leaf can appear in several layers of healthy leaves, lowering the quality of Chinese cabbage significantly [20]. Tipburn is caused by a variety of pathogenic variables, including soil physical and chemical properties, climatic circumstances, fertilizer, and others [20]. Tiburn in Chinese cabbage is a quantitative property controlled by numerous genes, according to new scientific research, and there are some differences in tipburn resistance among varieties [21]. As a result, selecting distinct cultivars to investigate the molecular mechanism of tipburn resistance genes is critical. The inbred lines ‘J95822’ and ‘J39290’ were used as research materials in this study. Through phenotypic observation, it was discovered that the phenotypes of the two varieties were similar (Fig. 1). At the same time, the incidence of tipburn was calculated, and the results showed that the tipburn resistance of ‘J39290’ was stronger than that of ‘J95822’ (Fig. 2). All these provide a good basis for studying the molecular mechanism of tipburn.

The accumulation of Ca2+ in the root of ‘J39290’ was higher than that of ‘J95822’ (Fig. 4C), as was the accumulation of Ca2+ in the interior leaves of ‘J39290’ (Fig. 4D). It was discovered that tipburn is associated to Ca2+ uptake and accumulation, which is in line with previous research [22]. In terms of Ca2+ concentration in interior leaves, there was no significant difference between ‘J39290’ and ‘J95822’ (Fig. 4A), which was not consistent with the previously reported results [23]. The explanation for this is that the concentration of Ca2+ in ‘J95822’ is not the main cause of Chinese cabbage tipburn. Furthermore, the amount of K+ accumulation in ‘J39290’ roots (56.50-57.80 mg/plant DW) was lower than in ‘J95822’ roots (84.75-98.87 mg/plant DW) (Fig. 4C), showing that the roots of ‘J39290’ boosted calcium uptake while decreasing potassium uptake. Tipburn may alter the uptake and distribution of other ions based on the concentration and accumulation of K+, Mg2+, and Na + in the roots, inside leaves, and outer leaves of tipburn sensitive and resistant Chinese cabbage (Fig. 4).

The intracellular Ca2+ homeostasis is controlled by Ca2+ storage and transport system [11]. Studies on Ca2+ transporters have also been reported in detail [10, 12, 13]. *ACA4* and *ACA11* are mostly expressed in *Arabidopsis thaliana* leaves and are found in the vacuole membrane.. The experimental results showed that *ACA4* and *ACA11* genes played an important role in transporting intracellular excess Ca2+ to vacuoles. After *ACA4* and *ACA11* double gene knockout mutations, scattered disease spots appeared around the leaves, especially the leaf margin [24]. Among the 11 CAX antiporters identified in *Arabidopsis thaliana* (*CAX1-11*), only *CAX1-4* has the activity of exchanging Ca2+/H+ in vacuoles [25–28]. *CAX2* and CAX4 can transport a series of cations, but do not play a major physiological role in Ca2+ homeostasis [28–30]. The double mutation of *CAX1*/*CAX3* showed necrosis of leaf tip and stem tip, and *CAX1* and *CAX3* antiporter could exchange one fine cytoplasm Ca2+ with two vacuole proton (H) [31]. ECA pumps (ECA1, ECA3 and ECA4) in intima are essential for the balance of Ca2+/Mn2+ between cytoplasm and endoplasmic reticulum [32–34]. These results suggest that ACA, CAX and ECA transporters are involved in the process of tipburn induced by Ca2+ deficiency.

Given the discovery of 7 *ECA* genes and 4 *CAS* genes in Chinese cabbage, the transcriptional patterns of these genes in the interior leaves of tipburn sensitive and resistant plants were investigated. Among the above 7 *ECA* genes, *Bra018690* (*ECA1*) was not expressed in the interior leaves, the FPKM of *Bra031593* (*ECA4*) and *Bra029645* (*ECA4*) showed opposite trends in the two materials, and there was no significant difference in the expression of other *ECA* genes between the two materials (Additional file 1: Table S5). Similarly, the expression levels of *Bra013013* (*CAS*) and *Bra003389* (*CAS*) in tipburn resistant Chinese cabbage plants were substantially greater than those in tipburn sensitive (Additional file 1: Table S5). The remaining *CAS* genes did not differ significantly between the two materials (Additional file 1: Table S5). The expression data of 7 *ECA* genes and 4 *CAS* genes in Chinese cabbage did not match the screening criterion of Fold Change≥2 and FDR < 0.01. As a result, we speculate that these genes aren’t solely responsible for inducing resistance in tipburn-resistant plants. *BrCRT2* is an important node gene in the interaction between Ca2+ pathway and tipburn resistance in Chinese cabbage, but we did not find this gene in the all DEGs. Based on the transcriptome sequencing results, we speculate that the plant ‘J39290’ protects the integrity of cell membrane not through calcium uptake and transport, but through other paths.

In this research, 23 tipburn associated genes were discovered using transcriptome analysis of tipburn sensitive and resistant Chinese cabbage (Fig. 6). The usage of qRT-PCR technique was also employed to confirm the accuracy of transcriptome sequencing results (Fig. 7C). According to various functional annotations and KEGG pathway predictions, a total of 23 differentially expressed genes participated plant-pathogen interaction pathway, and they encode three types of proteins: CDPK, Rboh, and CaM/CML proteins (Table 1; Additional file 1: Table S3). These proteins can participate in cell hypersensitivity response, cell wall reinforcement, and stomatal closure. Based on the above analysis, we speculated that the main cause of tipburn in Chinese cabbage ‘J95822’ was bacterial infection rather than lack of elements (calcium, etc.).

## Conclusions

We discovered tipburn resistant material ‘J39290’ and tipburn sensitive material ‘J95822’ after investigating the severity of tipburn and cell membrane structural integrity. The transcriptome data of the two samples was evaluated using DEG, GO, KEGG analysis, and protein interaction prediction. The findings revealed that 23 potential genes were detected in the ‘J39290’ inbred line’s tipburn resistance, and that these genes may promote cell integrity via the Ca2 + −CaM / CML-CDPK signal pathway, hence increasing tipburn resistance.

## Methods

### Plant materials

To explore the molecular mechanism of Chinese cabbage response to tipburn, we selected two inbred lines —‘J95822’ and ‘J39290’ to study. All of them are from the Vegetable Research Institute of Xinxiang Academy of Agricultural Sciences.

Seeds had been sown in seedlings medium (Metro Mix 350; Sun Gro Horticulture, Agawam, MA, USA) on August 20, 2020, and then cultured in the germination chamber at 26 °C and 70% humidity (16 h light/8 h dark cycle). Thirty plants of each variety were transplanted after one month later, and all plants in the field were managed uniformly in the process of growing. The incidence of tipburn in Chinese cabbage was analyzed at the rosette stage (November 12), the early heading stage (November 27) and the late heading stage (December 12). At the same time, the determination of nutritional elements in samples and the transcriptome sequencing were carried out at the late heading stage (December 12).

### Statistical method for the incidence of tipburn

We used “Overall incidence” and “Leaf incidence per plant” as indexes of tipburn to assess the severity of tipburn in Chinese cabbage. Overall incidence: the proportion of plants in one inbred line exhibiting tipburn symptoms to the total number of plants [35]. Leaf incidence per plant is the proportion of tipburn leaves to the total number of leaves of a plant.

### Determination method of nutrient content

In the late heading stage, the samples of outer leaves, interior leaves and roots of Chinese cabbage were sampled and the contents of K, Ca, Mg and Na were determined. The specific steps were as follows: (1) the collected samples were first killed at 105 °C, then dried in an oven at 70 °C, and finally crushed with a stainless steel grinder. (2) the dried sample (0.02-0.2 g) was weighed and filtered on the sieve of 0.15 mm. (3) the filtered sample was digested with mixed acid (HNO3: HClO4 = 3: 1), and the digested solution was fixed to 100 mL with water. (4) ICP-AES (Inductively coupled Plasma Emission Spectrometer) was used to determine the concentration of each element.

### The method of making leaf slices

To analyze the cellular structure of leaves in Chinese cabbage, we took the interior leaves of tipburn sensitive Chinese cabbage at the late heading stage to make leaf slices, including the critical areas of tipburn and non-tipburn. The same part of the tipburn resistance Chinese cabbage was used as control. The slices were placed in a pre-prepared FAA fixed solution (45% ethanol, 5% glacial acetic acid, 5% formaldehyde), stained with fuchsin (0.5%) and solid green (0.5%) solutions, dehydrated using an ethanol series, and sealed with neutral gum [36]. An OlympicBX51 microscope was used to examine and photograph the cell structure.

### Total RNA isolation, RNA-sequencing library construction and transcriptome sequencing

The interior leaves of tipburn resistant and tipburn sensitive Chinese cabbage were used to sequence the transcriptome (3 tipburn resistant samples, namely A1, A2, A3, and 3 tipburn sensitive samples, namely S1, S2, S3). To obtain good quality samples, we used a NanoDrop, a Qubit 2.0 and and an Agilent 2100 to test the purity, concentration and integrity of RNA. The process of library construction was as follows: we first (1) enrich eukaryotic mRNA; with oligo (dT) beads, (2) add fragmentation buffer to mRNA to randomly interrupt, (3) use mRNA as template, synthesize the first cDNA chain with six-base random primers (random hexamers), then add buffer, dNTPs, RNase H and DNA polymerase I to synthesize the second cDNA chain, and purify cDNA by AMPure XP beads. (4) The purified double-stranded cDNA was repaired by terminal repair, a tail was added and connected to the sequencing connector, and then the fragment size was selected by AMPure XP beads. (5) Finally, the cDNA library was obtained by PCR enrichment. The Q-PCR method was used to accurately determine the effective concentration of the library (the effective concentration of the library > 2 nM) to ensure the quality of the library. After passing the warehouse inspection, based on the Sequencing By Synthesis (SBS) technology of synthesis and sequencing, the sequencing was carried out on the Illumina platform.

### Sequencing data processing and DEG analysis

The data obtained from transcriptome sequencing were compared with Chinese cabbage genome sequence (Bra Chromosome V1.5) by HISAT2 2.0.4 efficient alignment system [37]. After the completion of the comparative analysis, the reads was assembled by StringTie [38], and compared with the original genome annotation information to find the annotated transcripts and the original unannotated transcripts, so as to explore the transcripts and genes of Chinese cabbage. The excavated genes were compared with NR [39], Swiss-Prot [40], GO [41], COG [42], KOG [43], Pfam [44] and KEGG database [45] by BLAST software [46]. The KEGG Orthology results of the gene were obtained by using KOBAS 2.0 [47]. To obtain gene annotation information, the predicted amino acid sequences were compared with Pfam database [48] by using Hmmer software.

Fragments per kilobase of transcript per million fragments mapped (FPKM) [49] was used as an index to measure the level of transcript or gene expression. We analyzed the differential expression among the sample groups by DESeq2 [50]. Fold Change ≥2 and FDR (False discovery rate) < 0.01 were selected as screening criteria. The fold change represents the ratio of expression between two groups. The FDR is obtained by correcting the significant difference using the *p*-value. Pearson correlation coefficient ‘*R2*’ [51] was used to analyze the correlation between the results of transcriptome sequencing and qRT-PCR.

### Verification of transcriptome sequencing results

To verify the reliability of the transcriptome sequencing results, the nucleotide sequences of 23 calcium-related DEGs were selected from the database, and specific primers were designed by Primer 5.0 [52] (Additional file 1: Table S4)**.** Actin (Gene ID: *Bra028615*) was used as an internal reference. Total RNA was first reverse-transcribed into cDNA using M-MLVRTase cDNA Synthesis Kit (Takara, Japan), and then analyzed the relative expression levels of 23 calcium-related DEGs on the ABI 7500 real-time PCR system with the Prime Script RT reagent kit (TaKaRa). The PCR system and PCR reaction conditions were set according to previous studies [53]. There were three biological and three technical replicates for each treatment. The comparative 2-ΔΔCT method [54] was used to analyze the relative expression profiles of genes. Statistical analysis was carried out by one-way ANOVA using SPSS 18.0 software. The data are presented as the mean ± SD. The relative expression levels of genes were demonstrated by HemI software [55].

### Analysis of protein interaction of candidate genes related to tipburn

We utilized STRING [56] to construct the DEG interaction network, combining the results of differential expression analysis with the interaction relationship pairings recorded in the database. The protein interaction network was visualized using the Cytoscape software [57].

## Supplementary Information

**Additional file 1: Table S1.** The data statistics of transcriptome sequencing. **Table S2.** Sequence alignment results between sample sequencing data and selected reference genome. **Table S3.** FPKM values and functional annotations of 23 genes involved in hypersensitive response, cell wall reinforcment and stomatal closure. **Table S4.** List of primer sequences used for qRT-PCR analysis of the tipburn related genes. **Table S5.** Transcriptional profile data of 7 *ECA* genes and 4 *CAS* genes in Chinese Cabbage.

**Additional file 2: Figure S1.** Principal component analysis of all genes in tipburn sensitive samples and tipburn resistant samples. **Figure S2.** FPKM and qRT-PCR values of 21 tipburn related protein genes.

**Additional file 3.**

## Data Availability

The datasets supporting the results described in this article were included within the article and its additional file, however, All raw sequence reads have been deposited in NCBI database, and the accession number is PRJNA743576.
