# Supplementary figures and images for: Identification of genes related to tipburn resistance in Chinese cabbage and preliminary exploration of its molecular mechanism
Source: BMC Plant Biol. 2021 Dec 3;21:567. doi: 10.1186/s12870-021-03303-z (PMC8641176; doi:10.1186/s12870-021-03303-z)

PCA

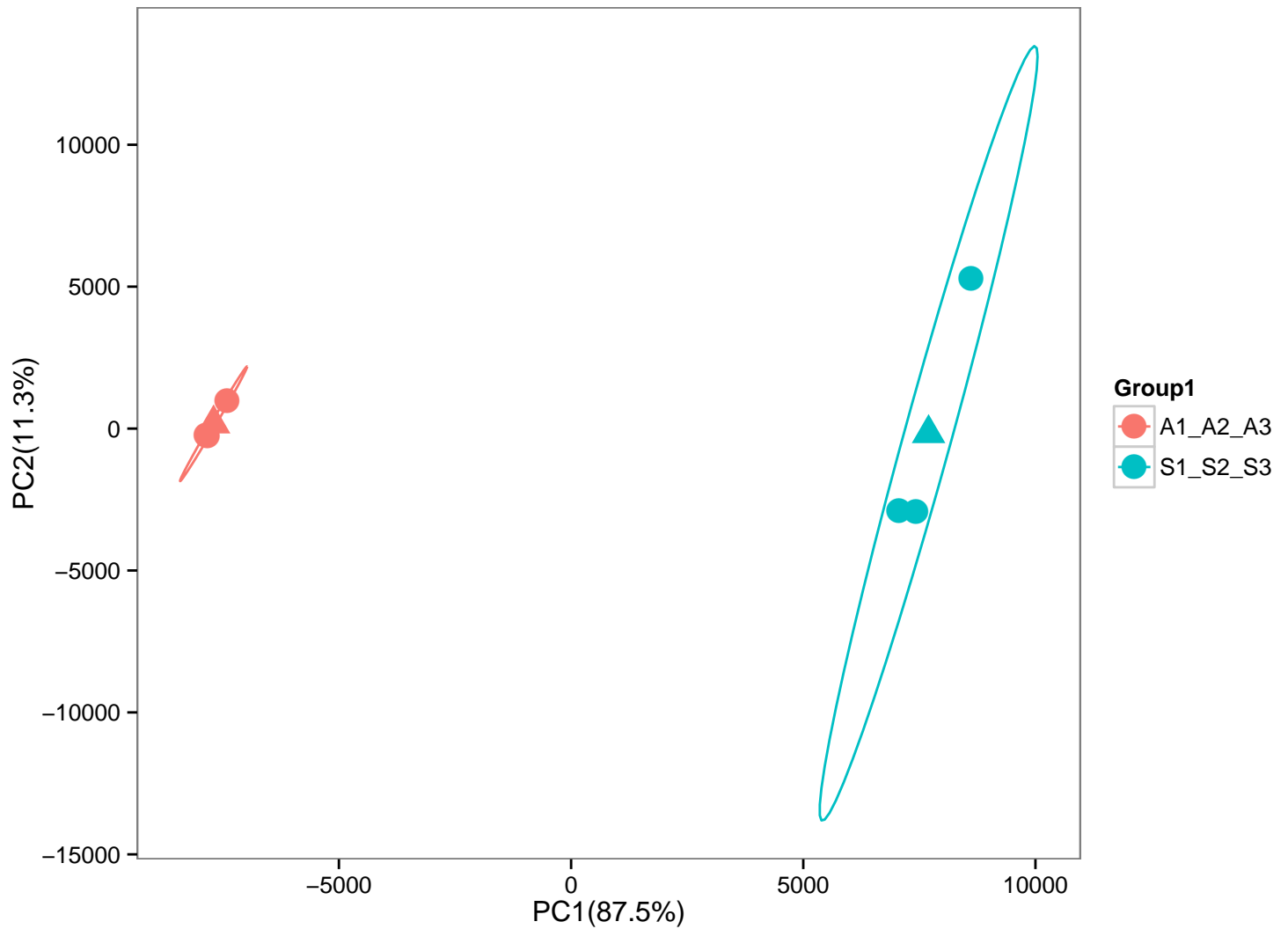

Supplement: Supplementary file 2 — Additional file 2: Figure S1. Principal component analysis of all genes in tipburn sensitive samples and tipburn resistant samples. Figure S2. FPKM and qRT-PCR values of 21 tipburn related protein genes. [file 12870_2021_3303_MOESM2_ESM.zip › Fig. S1.pdf]

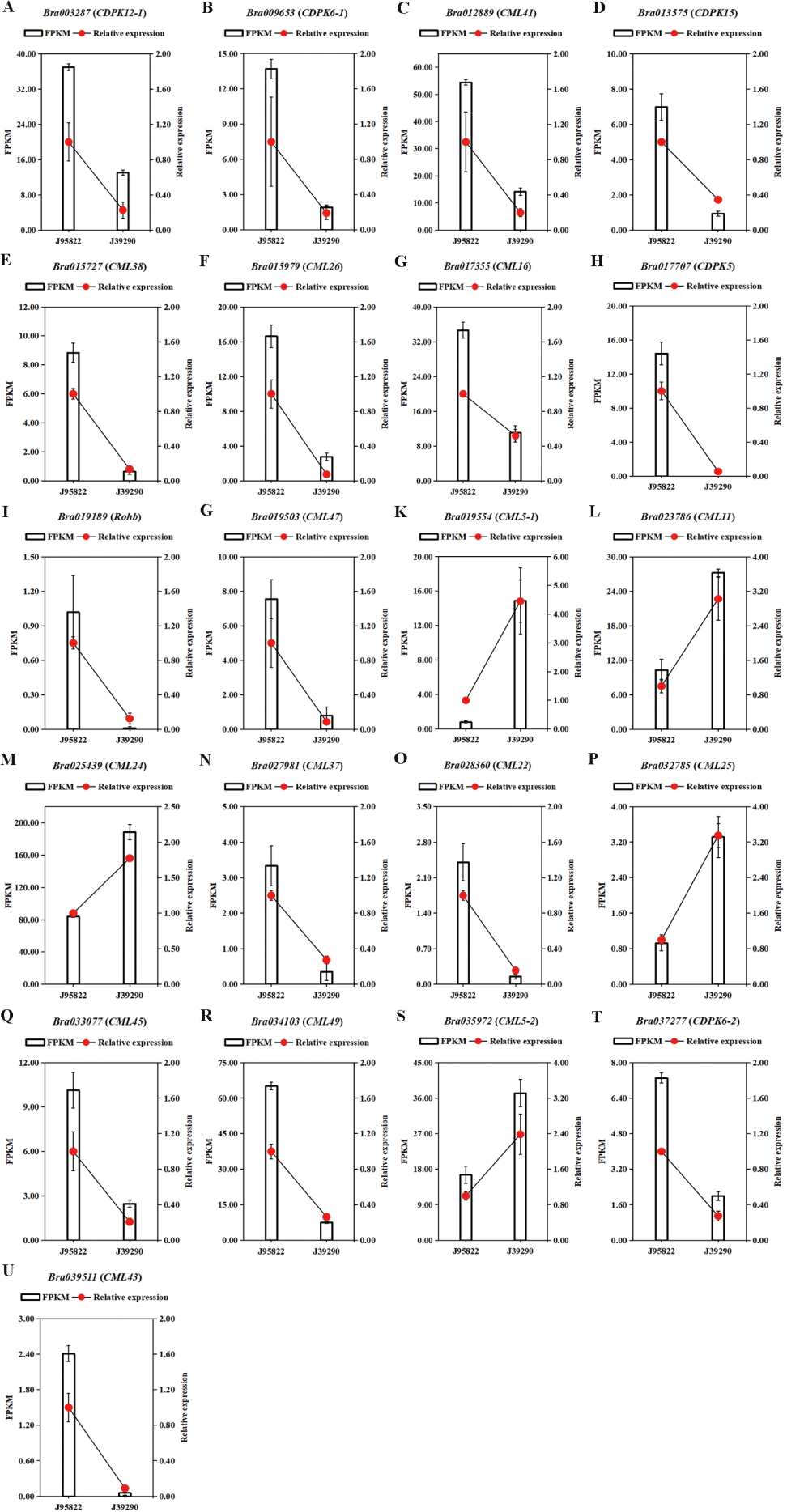

Supplement: Supplementary file 2 — Additional file 2: Figure S1. Principal component analysis of all genes in tipburn sensitive samples and tipburn resistant samples. Figure S2. FPKM and qRT-PCR values of 21 tipburn related protein genes. [file 12870_2021_3303_MOESM2_ESM.zip › Fig. S2.pdf]
